# Impact of mangrove forests degradation on biodiversity and ecosystem functioning

**DOI:** 10.1038/s41598-018-31683-0

**Published:** 2018-09-05

**Authors:** Laura Carugati, Beatrice Gatto, Eugenio Rastelli, Marco Lo Martire, Caterina Coral, Silvestro Greco, Roberto Danovaro

**Affiliations:** 1 Polytechnic University of Marche, Department of Life and Environmental Sciences, Ancona, 60131 Italy; 2 Stazione Zoologica Anton Dohrn, Naples, 80121 Italy

## Abstract

Mangroves are amongst the most productive marine ecosystems on Earth, providing a unique habitat opportunity for many species and key goods and services for human beings. Mangrove habitats are regressing at an alarming rate, due to direct anthropogenic impacts and global change. Here, in order to assess the effects of mangrove habitat degradation on benthic biodiversity and ecosystem functioning, we investigated meiofaunal biodiversity (as proxy of benthic biodiversity), benthic biomass and prokaryotic heterotrophic production (as proxies of ecosystem functioning) and trophic state in a disturbed and an undisturbed mangrove forests. We report here that disturbed mangrove area showed a loss of 20% of benthic biodiversity, with the local extinction of four Phyla (Cladocera, Kynorincha, Priapulida, Tanaidacea), a loss of 80% of microbial-mediated decomposition rates, of the benthic biomass and of the trophic resources. The results of this study strengthen the need to preserve mangrove forests and to restore those degraded to guarantee the provision of goods and services needed to support the biodiversity and functioning of wide portions of tropical ecosystems.

## Introduction

Mangrove ecosystems are of great ecological and economic importance^1^. They cover 15,000,000 ha^2^. with high biomass and economic values^3^. These forests, at the land-sea interface, provide food, breeding grounds and nursery sites for a variety of terrestrial and marine organisms, including many commercial species and juvenile reef fish^4,5^. Mangrove forests are highly productive ecosystems with rates of primary production equal to those of tropical humid evergreen forests^6^. They accumulate carbon in tree biomass, and most of this carbon is lost by decomposition and export to adjacent ecosystems^7^. Mangroves play also a key role in human sustainability and livelihoods, being heavily used for food, timber, fuel and medicine^8,9^. They offer protection from catastrophic events, such as tsunami, tropical cyclones and tidal bores and can dampen shoreline erosion^6,10^.

Despite their importance, mangroves are disappearing at a global loss rate of 1–2% per year^11^, and the loss rate reached 35% during the last 20 years^4,12^. Climate changes (sea level rise and altered rainfalls) and human activities (urban development, aquaculture, mining, and overexploitation of timber, fish, crustaceans and shellfish) represent major threats for mangrove habitats^13–16^.

Habitat loss is typically associated with a loss in terms of biodiversity^12^. Theoretical ecology predicts that biodiversity can influence ecosystems’ functioning, although outputs of correlative investigations and manipulative experiments have provided contrasting results^17^. The relationships between biodiversity and functioning of marine ecosystems are most often positive^18^, so that biodiversity loss could result in a reduction of the ecosystem functioning and, consequently, of the ecosystems’ capacity to provide goods and services to humans^19–22^. This is particularly evident in tropical ecosystems, such as mangroves, which host an important fraction of coastal biodiversity and are among those that will experience the earliest emergence of the impacts of global changes^23^. Sea level rise represents the main concern considering their tidal nature, but also changes in temperature, salinity, and increases in greenhouse gas concentrations need to be considered^3,10^. It has been reported that also changes in precipitations and thus in soil water content and salinity, can lead to variations in mangrove species composition and growth^10^.

In mangrove systems, a large proportion of the algal and leaf biomass are processed by searmid crabs, important keystone engineers in many forests^24,25^. In addition, in both sediments and tidal waters, organic matter and energy flow is funnelled through a highly diverse, actively growing, microbial loop and subsequently transferred to higher trophic levels through detritivorous, bacterivorous, and deposit feeders inhabiting the benthos^25,26^. Thus, a biodiversity loss in marine benthic biodiversity, whatever the phylum considered, could cause a variably reduction of ecosystem functions^26^.

Meiofauna are characterized by high abundance, species richness, short generation time and sensitivity to variations in environmental conditions^26,27^. In mangrove ecosystem, meiofaunal organisms play key ecological roles: i) accelerating re-mineralization of organic matter and thus nutrient regeneration, ii) stimulating prokaryotic activity and iii) sustaining mangrove food web^28–30^. All these characteristics, along with their direct contact with sediments as permanent members of the benthos, make them a potential tool for detecting rapid and unequivocal reaction of benthic assemblages to environmental changes.

**Figure 1:**
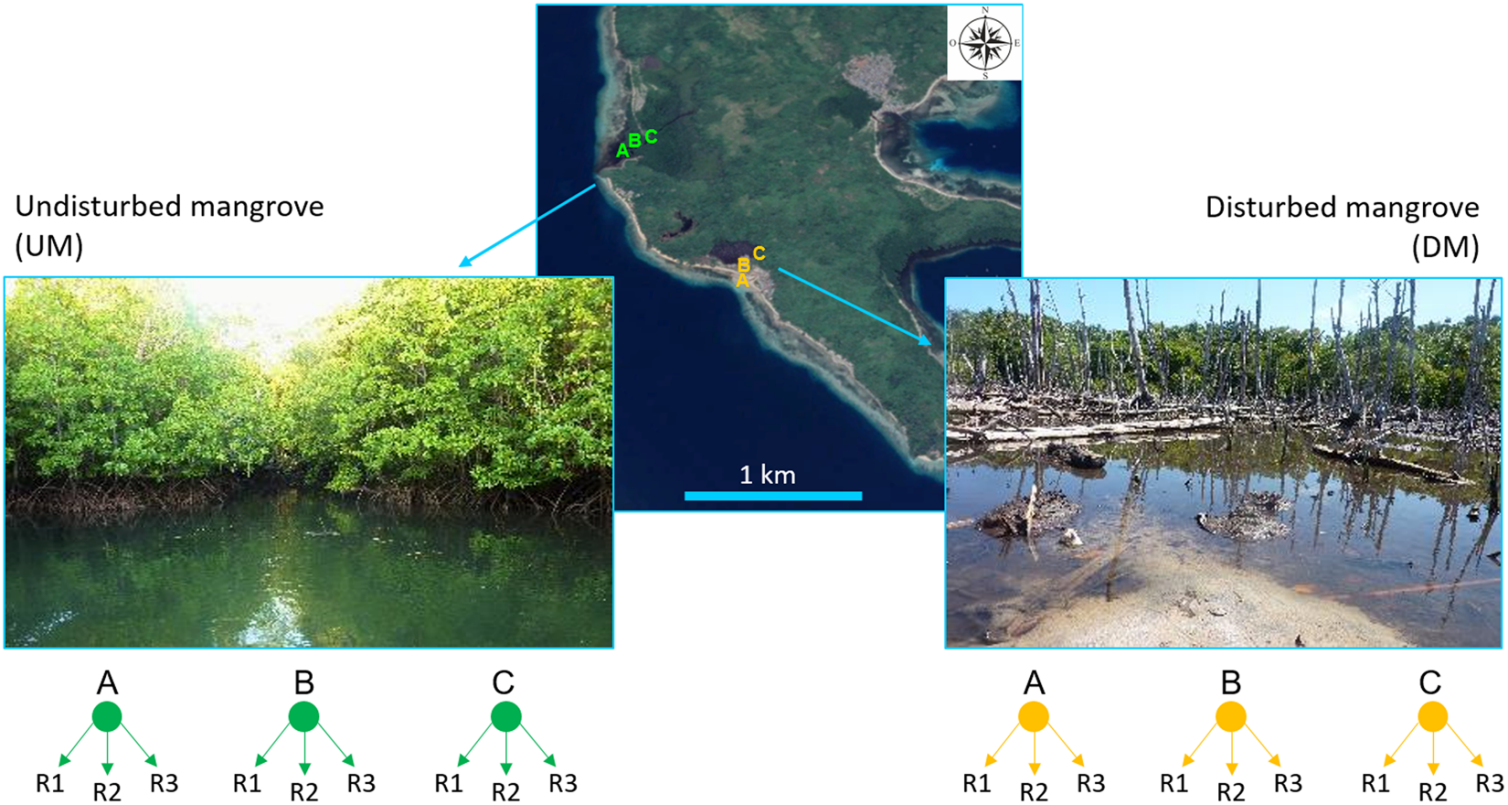
Sampling area and the location of the two investigated mangroves: Undisturbed Mangrove (UM) and Disturbed Mangrove (DM). Reported are sites (**A**–**C**) sampled within each mangrove area. The map was generated using Google Earth Pro (version 7.3.0.3832, 32-bit), https://earth.google.com (Map Data: Google, 2017 DigitalGlobe; Google, 2017 TerraMetrics; Google, 2017 CNES/Airbus), and modified using Microsoft Power Point (version 16.0.8201.2200, 32-bit).

In the present study, we investigated the effects of mangrove habitat degradation on trophic state and food availability, on biodiversity and on ecosystem processes by comparing an undisturbed with a disturbed mangrove forests (Fig. 1). We used meiofaunal biodiversity as a proxy of the overall benthic biodiversity, and benthic biomass and prokaryotic heterotrophic production (i.e., prokaryotic C incorporation) as proxies of ecosystem functioning. We hypothesised that disturbed mangrove area displays a lower biodiversity and altered ecosystem processes when compared to the undisturbed one.

## Results

Data on environmental variables (salinity, grain size) and on meiofaunal richness of taxa are reported in Table 1. In both mangrove systems, the redox potential discontinuity (RPD) level is ca. 2 cm below the sediment surface. The results of the PERMANOVA tests revealed the presence of significant differences between disturbed and undisturbed mangroves in most investigated variables (Tables 2–4).

**Table 1:** Area, site, salinity, grain size, meiofaunal richness of taxa in the sediments of the undisturbed and disturbed mangroves.

Area	Site	Salinity	Grain size	Meiofaunal taxa richness
				n
Undisturbed	A	32	Sand-mud	12
	B	30	Mud-sand	7
	C	28	Mud-sand	8
Disturbed	A	33	Sand-mud	8
	B	30	Mud-sand	7
	C	25	Very fine sand	6

**Table 2:** Output of the PERMANOVA analysis carried out to test for differences in total phytopigments, biopolymeric carbon, percentage of chlorophyll-a to biopolymeric carbon and to phytopigments, percentage of proteins to biopolymeric carbon, protein to carbohydrate ratio and biochemical composition of organic matter between undisturbed and disturbed mangrove areas (df = degrees of freedom; MS = mean square; Pseudo-F = F statistic; P(MC) = probability levels obtained from Monte Carlo asymptotic distributions).

Variable	Source	df	MS	Pseudo-F	P(MC)
Phytopigments	Area	1	9,05	21,15	**
	Site (Area)	4	0,70	1,65	ns
	Residual	12	0,43		
Biopolymeric C	Area	1	12,42	93,43	***
	Site (Area)	4	0,75	5,62	**
	Residual	12	0,13		
Chlorophyll-a to biopolymeric C %	Area	1	5,91	81,05	***
	Site (Area)	4	2,55	35,01	***
	Residual	12	0,07		
Chlorophyll-a to phytopigments %	Area	1	4,05	10,70	**
	Site (Area)	4	2,10	5,57	**
	Residual	12	0,38		
Protein to biopolymeric C %	Area	1	7,65	67,36	***
	Site (Area)	4	2,00	17,60	***
	Residual	12	0,11		
Protein to carbohydrate ratio	Area	1	5,90	96,43	***
	Site (Area)	4	2,59	42,37	***
	Residual	12	0,06		
Biochemical composition	Area	1	43,77	28,80	***
	Site (Area)	4	5,75	3,78	**
	Residual	12	1,52		
	Total	17			

***P < 0.001; **P < 0.01; ns = not significant.

**Table 3:** Output of the PERMANOVA analysis carried out to test for differences in total meiofaunal abundance, richness of higher taxa, taxonomic composition between undisturbed and disturbed mangrove areas (df = degrees of freedom; MS = mean square; Pseudo-F = F statistic; P(MC) = probability levels obtained from Monte Carlo asymptotic distributions).

Variable	Source	df	MS	Pseudo-F	P(MC)
Abundance	Area	1	5,16E + 06	4,35	*
	Site (Area)	4	6,64E + 06	5,60	**
	Residual	12	1,19E + 06		
Richness of higher taxa	Area	1	8,00	6,26	*
	Site (Area)	4	4,94	3,87	*
	Residual	12	1,28		
Composition as higher taxa	Area	1	1527,60	1,51	ns
	Site (Area)	4	2768,10	2,74	**
	Residual	12	1010,50		
Composition as rare taxa	Area	1	9352,90	5,51	**
	Site (Area)	4	4453,10	2,62	**
	Residual	12	1697,30		
	Total	17			

**P < 0.01; *P < 0.05; ns = not significant.

**Table 4:** Output of the PERMANOVA analysis carried out to test for differences in prokaryotic biomass and heterotrophic production between undisturbed and disturbed mangrove areas (df = degrees of freedom; MS = mean square; Pseudo-F = F statistic; P(MC) = probability levels obtained from Monte Carlo asymptotic distributions).

Variable	Source	df	MS	Pseudo-F	P(MC)
Prokaryotic biomass	Area	1	13,38	824,49	***
	Site (Area)	4	0,86	52,72	***
	Residual	12	0,02		
Heterotrophic production	Area	1	10,12	135,72	***
	Site (Area)	4	1,50	20,04	***
	Residual	12	0,07		
	Total	17			

***P < 0.001.

### Sedimentary variables

**Figure 2:**
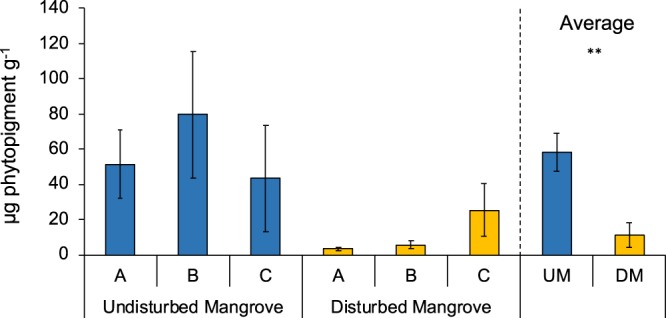
Total phytopigments. Reported are the concentrations of phytopigments in undisturbed and disturbed mangrove areas. Reported are also average values of Undisturbed Mangrove (UM) and Disturbed Mangrove (DM) ± standard error.

The results of the PERMANOVA carried out between the two mangroves revealed the presence of significant differences for quantity and quality of organic matter (OM) (Table 2). The sedimentary concentrations of chlorophyll-a and total phytopigments were significantly higher in the undisturbed mangrove than in the disturbed one (PERMANOVA, P < 0.01; Fig. 2; Table 2). Chlorophyll-a was four times lower in the disturbed forest (3 ± 1 µg g^−1^) than in the undisturbed one (12 ± 2 µg g^−1^), whereas phytopigments were five times higher in the sediments of the undisturbed area (58 ± 11 µg g^−1^) than in the sediments of the disturbed one (11 ± 7 µg g^−1^). In the undisturbed mangrove, total phytopigments picked at site B (80 ± 36 µg g^−1^) and were lower at site C (44 ± 30 µg g^−1^). In the sediments of disturbed forest, concentration of phytopigments ranged from 3 ± 1 µg g^−1^ at site A to 26 ± 15 µg g^−1^ at site C.

**Figure 3:**
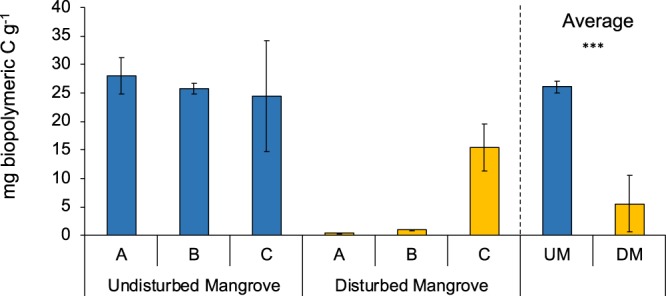
Biopolymeric carbon. Reported are the concentrations of biopolymeric carbon in undisturbed and disturbed mangrove areas. Reported are also average values of Undisturbed Mangrove (UM) and Disturbed Mangrove (DM) ± standard error.

The quantity of sedimentary organic matter, in terms of proteins, carbohydrates, lipids, were significantly higher in the sediments of undisturbed mangrove than in the disturbed one (PERMANOVA P < 0.001; Supplementary Figure S1). The concentrations of biopolymeric C was five times higher in the undisturbed (26 ± 1 mg g^−1^) than in the disturbed forest (6 ± 4 mg g^−1^) (PERMANOVA, P < 0.001; Fig. 3; Table 2). In the undisturbed area, biopolymeric C ranged from 28 ± 3 mg g^−1^ at site A to 24 ± 10 mg g^−1^ at site C. Whereas, in the disturbed area, sedimentary concentrations of biopolymeric C varied from 0.4 ± 0.1 mg g^−1^ at site A to 15 ± 4 mg g^−1^ at site C (Supplementary Table S1).

In both the undisturbed and disturbed mangroves, carbohydrate carbon represented the major fraction of biopolymeric C, but at different extend, accounting on average for 68 and 42%, in undisturbed and disturbed mangroves, respectively. Protein carbon represented on average 21% in the undisturbed forest and 42% in the disturbed one. Lipids accounted at a similar percentage in both the areas, representing on average 9 and 8% of biopolymeric C, in the undisturbed and disturbed forests, respectively. Protein fraction of biopolymeric C was double in the disturbed than undisturbed mangrove area and values of the protein to carbohydrate ratio were four times significantly higher in the sediments of disturbed mangrove than in those of the undisturbed one (PERMANOVA, P < 0.001; Table 2).

### Faunal diversity and assemblage structure

**Figure 4:**
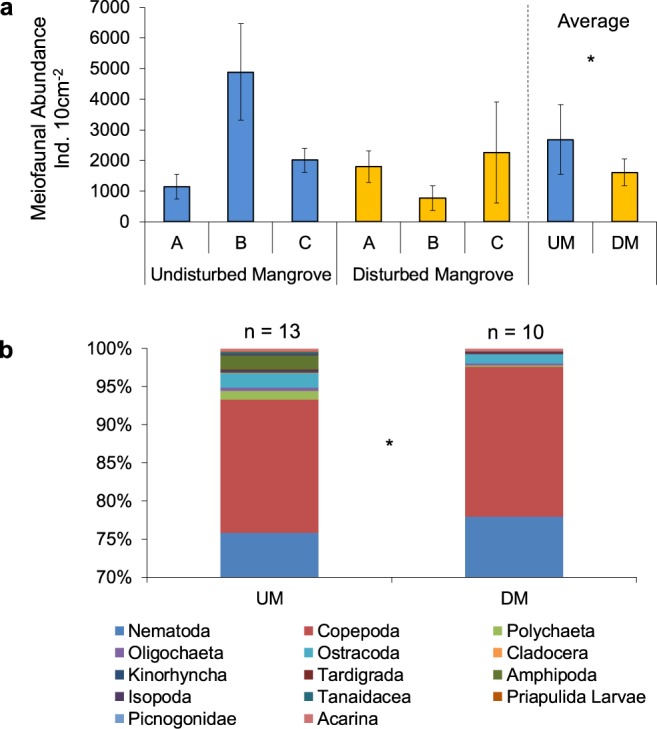
Meiofaunal assemblages. Illustrated are meiofaunal abundance (**a**) and taxonomic composition (**b**) with the number of higher taxa found in the sediments of undisturbed and disturbed mangroves. Reported are also average values of Undisturbed Mangrove (UM) and Disturbed Mangrove (DM) ± standard error.

Data on meiofaunal abundance, richness of taxa and taxonomic composition are shown in Fig. 4a,b. Meiofaunal abundance was significantly higher in the sediments of undisturbed mangroves (2684 ± 1132 ind. 10 cm^−2^) than in the sediments of disturbed ones (1614 ± 441 ind. 10 cm^−2^) (PERMANOVA, P < 0.05; Fig. 4a; Table 3). In the undisturbed mangrove area, the total number of meiofaunal individuals was higher at site B (4893 ± 1572 ind. 10 cm^−2^) than at site A (1148 ± 401 ind. 10 cm^−2^) and C (2012 ± 389 ind. 10 cm^−2^). In the disturbed forest, the highest value of meiofaunal abundance was recorded in sediments at site C (2266 ± 1651 ind. 10 cm^−2^), whereas the lowest one was found at site B (775 ± 402 ind. 10 cm^−2^) (Supplementary Table S2).

Overall, 14 taxa have been identified in the two sampling areas, and PERMANOVA tests revealed that the richness of meiofaunal taxa was significantly higher in the sediments of undisturbed mangrove (13 taxa) than in those of disturbed area (10 taxa) (PERMANOVA, P < 0.05; Fig. 4b; Table 3). In both areas and at all sites, nematodes were the dominant taxon (76 and 78% in the undisturbed and disturbed mangroves, respectively), followed by copepods (18 and 20%) and ostracods (2% in both areas). The contribution of all other identified taxa (acarins, amphipods, cladocerans, isopods, kinorinchs, oligochaetes, tanaidaceans, tardigrades, priapulids larvae, pycnogonids, polychaetes) varied from 0 to 11% of the total meiofaunal abundance (Fig. 4b). Amphipods, isopods, oligochaetes, polychaetes, tardigrades were encountered in both sampling areas. Cladocerans, kinorinchs, priapulids larvae, tanaidaceans occurred exclusively in the undisturbed mangrove area, whereas pycnogonids were observed only in the sediments of disturbed one, at site B.

**Figure 5:**
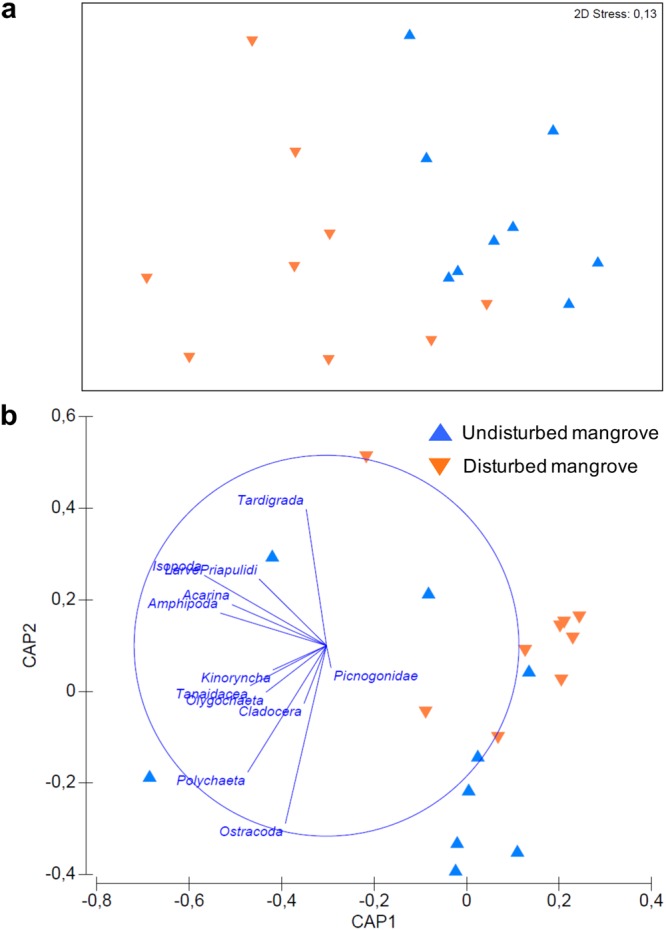
Taxonomic composition of rare meiofaunal taxa. MDS ordination plot (**a**) and output of canonical analysis of principal coordinates (CAP) (**b**) illustrating the differences in the composition of meiofaunal assemblages (excluding nematodes and copepods) in the sediments of the two investigated areas.

The taxonomic composition of meiofaunal higher taxa did not significantly vary between the two mangroves (PERMANOVA, ns; Table 3). Nevertheless, the results of the pairwise tests showed that the meiofaunal assemblages significantly changed between sites sampled in the undisturbed mangroves (Supplementary Table S2). The taxonomic composition of rare meiofaunal taxa (i.e., excluding nematodes and copepods) varied significantly between the sediments of the undisturbed and disturbed mangroves (PERMANOVA, P < 0.01). This has been confirmed also by the Multi-Dimensional Scaling (MDS) plot and the results of the Canonical Analysis of Principal Coordinates (CAP) analyses (Fig. 5a,b).

The SIMPER analysis revealed that the highest dissimilarity in the meiofaunal assemblage occurred among sites in the undisturbed mangrove (52%) than that among the two forests (49%). Whereas, the meiofaunal beta diversity of rare taxa was higher between the two sampling forests (78%) and lower values were found comparing sites among the same sampling area (37% in the disturbed forest and 53% in the undisturbed one). Variations in ostracods and polychaetes abundance were responsible for the observed percentage dissimilarity, as also shown in the plots of canonical analysis of principal coordinates (Fig. 5b).

### Biomasses and processes

**Figure 6:**
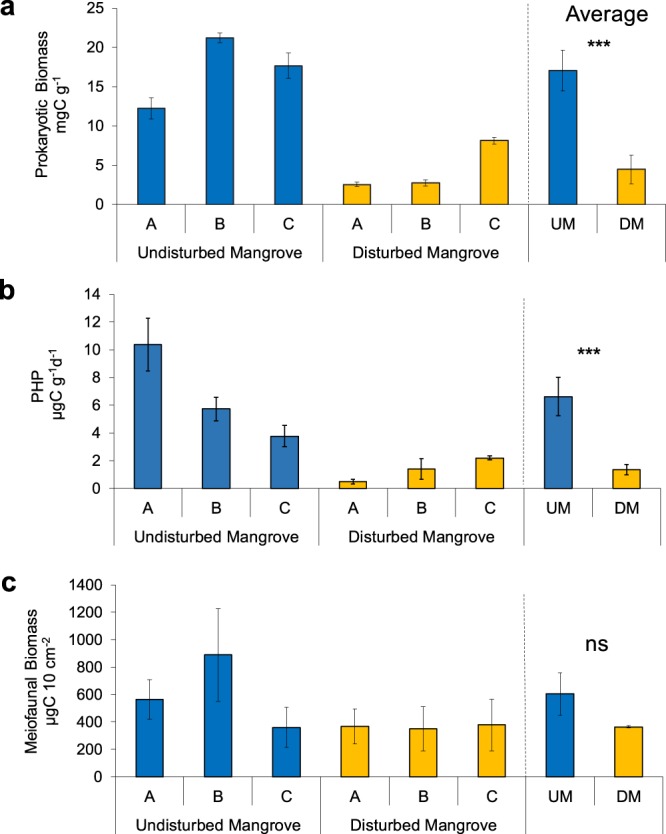
Ecosystem processes. Illustrated are prokaryotic biomass (**a**), prokaryotic heterotrophic production (µgC g^−1^ d^−1^) (**b**) and meiofaunal biomass (**c**) in undisturbed and disturbed mangrove areas. Reported are also average values of Undisturbed Mangrove (UM) and Disturbed Mangrove (DM) ± standard error.

Prokaryotic biomass was significantly higher in the undisturbed area (17 ± 3 µgC g^−1^) than in the disturbed one (5 ± 2 µgC g^−1^). In the undisturbed forest, prokaryotic biomass showed the highest value at site B (21.2 ± 0.6 µgC g^−1^) and the lowest at site A (12 ± 1 µgC g^−1^). In the disturbed mangrove area, prokaryotic biomass showed lower values in sediments at site A (2.6 ± 0.3 µgC g^−1^) and higher values in sediments at site C (8.1 ± 0.4 µgC g^−1^) (Supplementary Table S3). Prokaryotic heterotrophic production (PHP) were significantly higher in the undisturbed mangrove (7 ± 1 µgC g^−1^ d^−1^) than in the disturbed one (1.4 ± 0.4 µgC g^−1^ d^−1^) (PERMANOVA, P < 0.001; Fig. 6a,b; Table 4). In the undisturbed mangrove area, PHP values varied from 3.8 ± 0.8 to 10 ± 2 µgC g^−1^ d^−1^, at site C and A, respectively. In the disturbed mangrove, values of PHP ranged from 0.5 ± 0.2 to 2.2 ± 0.1 µgC g^−1^ d^−1^, at site A and C, respectively (Supplementary Table S3).

Meiofaunal biomass showed double values in the sediments of the undisturbed forest (604 ± 154 µgC 10 cm^−2^), than in the disturbed one (364 ± 8 µgC 10 cm^−2^), but they did not significantly vary (Fig. 6c; PERMANOVA, ns). In undisturbed mangrove, values of meiofaunal biomass ranged from 360 ± 147 µgC 10 cm^−2^ in sediments at site C to 888 ± 339 µgC 10 cm^−2^ in sediments at site B. In the disturbed forest, meiofaunal biomass varied from 351 ± 161 µgC 10 cm^−2^ in sediments at site B, to 377 ± 189 µgC 10 cm^−2^ in sediments at site C.

## Discussion

### Effect of habitat degradation on trophic state and food availability

In the present study, we found significant differences between the undisturbed and disturbed mangrove areas in terms of quantity and quality of sedimentary organic matter. In the sediments of undisturbed mangrove, the concentration of biopolymeric carbon and total phytopigments, which fall within the range of previous studies^26,31,32^, were ca 5 times higher than those reported for the sediments of disturbed mangrove area. Our results provide evidence that the main component of OM in mangrove habitat was represented by carbohydrates that usually dominate in all vegetated systems, representing up to 66% of organic carbon in plants^26,33^. The values of components of organic matter (i.e., proteins, carbohydrates and lipids) as well as the indicators of freshly produced autotrophic biomass (i.e., chlorophyll-a and phaeopigments), which could be the basis of the benthic food webs and sustain the trophic guild of detritus feeders, were several times higher in the sediments of undisturbed mangrove than in those of the disturbed one. The higher proteins:carbohydrates ratio found in the disturbed area could be driven by complex interactions with environmental conditions and biological processes constraining the degradation of proteins. Indeed, it has been recently demonstrated that some labile compounds (i.e., proteins or sugars) can persist not for weeks but for decades because of the requirement of co-metabolism with missing compound, or the presence of microenvironmental conditions that restrict the access (or activity) of enzymes^34^. Our results clearly indicate that the degradation of the mangrove habitat determined a collapse of the ability of these systems to produce OM. Although this finding was expected, we are now in the position to provide direct evidence that the ability to store organic material in surface sediments was reduced by ca 80% in the disturbed forest when compared to the undisturbed one.

### The effects of mangrove habitat degradation on biodiversity

Mangrove sediments usually host a significantly lower meiofaunal abundance when compared to the adjacent soft bottoms systems^26,30,35^. These differences are generally related to the huge organic enrichment leading to the confinement of the fauna in the top few oxygenated mm of the sediments^36^. In the present study the lower meiofaunal abundance and diversity we found in the disturbed area cannot be explained by oxygen availability (since the sediments of disturbed mangrove displayed similar oxygen penetration in the sediments) and were likely linked to the extreme conditions (higher temperatures and irradiation) characterizing the disturbed area as well as to the lower organic matter availability.

Moreover, we here report that meiofaunal diversity (in terms of higher taxa) was significantly lower in the disturbed than in the undisturbed mangrove sediments. The dissimilarity between the undisturbed and disturbed sampling areas was related to the loss, in the latter, of Cladocera, Kynorincha, Priapulida and Tanaidacea, which are known to be sensitive to the changes determined by habitat loss^37^. Some of these taxa, indeed, display habitat preference for the vegetated systems and the colonization/utilization of vegetal debris^37^. Kynorincha have been also suggested as sentinel of impact, as they disappear in altered or contaminated sediments^38,39^.

In addition, the undisturbed mangrove area was characterised by a higher spatial variability (as indicated by higher beta diversity found among sites). This finding reflects the presence of several types of substrates, even at smaller spatial scale (tens of cm), such as bare sediments at different decomposition stages, leaf litter and biotic surfaces (e.g., aerial roots, pneumatophores), which lead to the presence of different microenvironments, supporting a more diverse fauna^40,41^. Such a variability at small spatial scale is common in soft bottom ecosystems, which are typically characterized by high variability in environmental variables, even at the scale of few centimetres^42^. Overall these findings suggest that habitat degradation led to an average reduction of ca 40% of the abundance of individuals and, at the level of higher taxa, a loss of biodiversity of ca 20%.

### Effects of habitat degradation on ecosystem processes

In the present study, we utilised 3 main proxies of ecosystem functioning: prokaryotic biomass, heterotrophic production and meiofaunal biomass, which reflect the ability of the system to perform organic matter degradation and to convert primary production in biomass^20^. In the disturbed mangrove area, the values of prokaryotic biomass were three times lower than those observed in the undisturbed one. Similarly, prokaryotic heterotrophic production was 5 times lower in the sediments of disturbed mangroves. Meiofaunal biomass reflects the accumulation of organic detritus, the concentrations of labile organic compounds and of vegetal biomass (expressed as concentration of total phytopigments). Higher values of meiofaunal biomass were observed at all sites sampled in the undisturbed area.

Such differences suggest that disturbed sediments can loss ca 80% of their potential to degrade/utilise carbon resources and ca 40% of faunal biomass, when compared to undisturbed ones.

## Conclusions

Overall, our results indicate that the sediments of disturbed mangroves, when compared to undisturbed ones, were characterized by altered biogeochemical cycles and a different diagenesis of the organic matter, as pointed out by the significant decrease of sedimentary organic carbon, the potential of OM degradation by microbial metabolism, biomass and biodiversity of meiobenthic assemblages. Since meiofaunal biomass is the main target for the feeding of juvenile reef fishes that are particularly abundant in all mangrove systems^37,43^, these findings indicate that mangrove degradation could have important consequences also on neighbouring ecosystems and functions. Our study highlights the need of further understanding the effects of anthropogenic and natural stressors on mangrove ecosystems. Additional efforts are also needed to manage human activities within mangrove catchment, to conserve and sustainably use mangroves and, in case of habitat loss, to restore such important ecosystems, in order to ensure the provision of goods and services, and related ecological and economic benefits they provide.

## Methods

### Study area

This study has been conducted in a small archipelago located at latitude 1°45’ N (Fig. 1; Table 1). The investigated equatorial region hosts different marine ecosystems spanning from mangrove forests to seagrass meadows. The archipelago is impacted by different anthropogenic activities including destructive fishing (e.g., blast fishing and poison fishing) and kind of exploitation of the natural resources. Human impacts in the last years have determined the rapid degradation of wide portions of the mangroves of the island, while other remain pristine and were selected for a comparison (Supplementary Study area).

### Sampling strategy

Two sampling areas were compared in this study. The first one is represented by an undisturbed mangrove forest, located distant from human settlements. It was dominated by *Rhizophora sp*., while *Sonneratia alba* and *Bruguiera spp*. were less abundant. The undisturbed area of study was supplied with salt/brackish water from the tide. Some scuba diving and few fishing activities were observed, but there was no evidence of disturbance occurring and the mangroves were not affected. The disturbed area was located near to a local village and characterized by desiccated and dead mangroves. It was dominated by red mangroves, as the undisturbed forest. The disturbed area was affected by anthropogenic activities, i.e., tree cutting, housing settlement, sewages and fishing activities. In both sampling areas, three sites (A, B, C) were selected according to a stratified random sampling design (Fig. 1, Table 1). All sediment samples have been collected by using Plexiglas manual cores (inner diameter 3.6 cm). At each site in each mangrove area, three replicate sediment samples were collected for organic matter and prokaryotic analyses and three replicates were collected for meiofaunal analyses. Most of sampling sites presented comparable characteristics in terms of grain size (mud-sand and sand-mud; Table 1) and sedimentary vertical profile in terms of the depth of the RPD level (ca. 2–3 cm). All sediment samples for the determinations of OM, meiofaunal and prokaryotic assemblages were stored at −20 °C until the analyses in the laboratory, whereas samples for the determination of prokaryotic heterotrophic production were immediately incubated as described below. Despite the storage at −20 °C, all the identified organisms, including the soft-body individuals, resulted well-preserved. In addition, freezing did not damage the morphological features used to recognise organisms at the higher taxonomic levels (order, class or phylum) to which we identified them.

### Sedimentary organic matter

Once at laboratory, sediment samples were analysed for OM biochemical composition in terms of phytopigment (chlorophyll-a and phaeopigments), protein, carbohydrate and lipid contents. Proxies of primary organic material associated with primary producers, namely chlorophyll-a and phaeopigments were analysed fluorometrically^44^. Chlorophyll-a and phaeopigment concentrations were summed up and reported as total phytopigment (CPE) concentrations. Total phytopigment contents were utilized as an estimate of the organic material of algal origin, including the living (chlorophyll-a) and senescent/detrital (i.e., phaeopigments) fractions and converted into C equivalents^33,45^. Protein, carbohydrate and lipid contents were determined spectrophotometrically^33,45^. The concentrations were converted to C equivalents and their sum referred as biopolymeric C, BPC^33,45^.

The percentage contributions of chlorophyll-a to biopolymeric C concentrations and the values of the protein to carbohydrate ratio were then used as descriptors of ageing and nutritional quality of OM in the sediment^33^. The percentage contribution of total chlorophyll-a to biopolymeric C is an estimate of the freshness of the organic material deposited in the sediment: since photosynthetic pigments and their degradation products are assumed to be labile compounds in a trophodynamic perspective, the lower their contribution to sediment organic C the more aged the organic material^46^. Since N is the most limiting factor for heterotrophic nutrition and proteins are N-rich products, the protein to biopolymeric C and the protein to carbohydrate ratios are indicative of the nutritional value of the organic matter^33,46^.

### Prokaryotic abundance and biomass

Total prokaryotic abundance was determined by epifluorescence microscopy^47^. Sediment samples were treated three times for 1 min by ultrasounds (Branson Sonifier 2200, 60 W) after addition of 0.2 µm pre-filtered tetrasodium pyrophosphate solution at a final concentration of 5 mM, then properly diluted before filtration onto 0.2 µm pore-size Nuclepore black filters (Whatman). Each filter was then stained with 20 µl of SYBR Green I (Sigma Chemicals, previously diluted 1:20 with 0.2 µm pre-filtered Milli-Q water), washed twice with 3 ml sterilized Milli-Q water and mounted onto microscope slide. Filters were analyzed using epifluorescence microscopy (Zeiss Axioskop 2MOT, magnification 1,000×). At least 20 microscope fields and 400 cells were respectively observed and counted for each filter^48^. Prokaryotic abundance was expressed as cells per g of dry sediment, after desiccation at 60 °C for 24 h^45^. Prokaryotic biomass was determined based on cell size, converted into bio-volume, assuming 310 fg C$\,{\rm{\mu }}$ m^3^ as a conversion factor, following standard inter-calibration with Scanning Electron Microscope (SEM)^45,48,49^.

### Prokaryotic Heterotrophic Production

^3^[H]–leucine incorporation method was used for the determination of PHP, according to the procedure previously described^45,48,50^. Sediment samples were added with 0.2-µm pre-filtered seawater, containing ³[H]-leucine (68 Ci mmol^−1^; final 0.5–1.0 µM), then incubated in the dark, at *in-situ* temperature. To define the linearity and the saturation level of the ³[H]-leucine incorporation, time-course experiments over 6 h and concentration-dependent incorporation experiments (from 0.05 μM to 5.0 μM leucine) were also carried out. Blanks (n = 3) for each sediment sample were added with ethanol immediately before^3^[H]-leucine addition. After incubation, samples were supplemented with ethanol (80%), centrifuged, washed again two times with ethanol (80%), and the sediment was re-suspended in ethanol (80%) and filtered onto polycarbonate filters (0.2 µm pore size; vacuum <100 mm Hg). Afterward, each filter was washed four times with 2 ml of 5% TCA, then transferred into a Pyrex tube containing 2 ml of NaOH (2 M) and incubated for 2 h at 100 °C. After centrifugation at 800 × g, 1 ml of supernatant fluid was transferred to vials containing the appropriate scintillation liquid. A liquid scintillation counter (PerkinElmer-Packard Tri-Carb 2100 TR) was used to measure the incorporated radioactivity in the sediment samples^48,50^. The prokaryotic heterotrophic production was calculated by equation (1):

1 $${\rm{Prokaryotic}}\,{\rm{heterotrophic}}\,{\rm{production}}={\rm{LI}}\times 131.2\times {( \% {\rm{Leu}})}^{-1}\times ({\rm{C}}/{\rm{protein}})\times {\rm{ID}}$$

where: LI is the leucine incorporation rate (mol g^−1^ h^−1^), 131.2 is the molecular weight of leucine, %Leu is the fraction of leucine in a protein (0.073), C/protein is the ratio of cellular carbon to protein (0.86), and ID is the isotope dilution, assuming a value of 2.

### Meiofaunal abundance, taxon diversity and biomass

Each sediment sample was treated with ultrasound (for 1 min 3 times, with 30 s intervals) to detach organisms from the grain particle surface and, then, carefully and gently sieved through a 1000-µm and a 20-µm mesh net to retain the smallest organisms. The fraction remaining on the latter sieve was re-suspended and centrifuged three times with Ludox HS 40 (final density of 1.18 g cm^−3^)^51^. Subsequently, sediment samples have been carefully checked to search for remnant organisms. After staining with Rose Bengal (0.5 gL^−1^), all specimens were counted and classified per taxon, under a stereomicroscope, using a Delfuss cuvette^26^. Meiofaunal taxa representing <1% of the total meiofaunal abundance were defined as rare taxa^52^. Meiofaunal biomass was assessed by bio-volumetric measurements of all retrieved specimens. Nematode biomass was calculated from their biovolume, using the Andrassy’s^53^ formula (V = L × W^2^ × 0.063 × 10^−5^, in which body length, L, and width, W, are expressed in µm). Body volumes of all other taxa were derived from measurements of body length (L, in mm) and width (W, in mm), using the formula V = L × W^2^ × C, where C is the conversion factor specific for each meiofaunal taxon, used to convert L × W^2^ to body volume, according to models relating body dimensions and volume^54^. Each body volume was multiplied by an average density of 1.13 g cm^−3^ to obtain the biomass. The carbon content was considered to be 40% of the dry weight^54^.

### Statistical analyses

To assess differences between the two mangrove areas and sites, we applied uni- and multivariate distance-based permutational analyses of variance (PERMANOVA). All the statistical analyses were carried out using the same sampling design, considering two factors as main sources of variance: *Area* (fixed, two levels: undisturbed and disturbed mangroves) and *Site* (fixed, three levels: A, B, C, nested in *Area*).

Univariate distance-based permutational analyses of variance (PERMANOVA) were used to assess the variability in the OM compounds contents, total meiofaunal abundance and biomass, prokaryotic biomass and heterotrophic production^55,56^. The variability in the biochemical composition and nutritional quality of OM, taxonomic composition of meiofaunal communities were assessed using distance-based permutational multivariate analyses of variance (PERMANOVA). The analyses were carried out on Euclidean distances (for organic matter, prokaryotic and meiofaunal abundance and biomass) or Bray–Curtis similarity matrices (for meiofaunal taxonomic composition) of previously normalized (OM) or untransformed (faunal) data, using 999 permutations of the residuals under a reduced model. Bray-Curtis distance matrix was used for meiofaunal taxonomic composition, because for differences in community structure and composition, the semi-metric Bray–Curtis measure^57^ of ecological distance is preferred over metric measure^55^, like Euclidean distance^57–61^. Significant differences were investigated using *a posteriori* pair-wise test. P values in the PERMANOVA and pairwise tests were obtained from Monte Carlo asymptotic distributions, because of the restricted number of unique permutations^62^.

To visualize differences between areas in the meiofaunal community, Multidimensional scaling (MDS) and bi-plots after a CAP were prepared^63^.

To assess the percentage of dissimilarity^64^ in the meiofaunal assemblage composition among the sampling areas for (i) higher taxa and (ii) rare taxa and to identify the meiofaunal taxa most responsible for the observed differences, SIMPER analyses were carried out. A ranked matrix of Bray–Curtis similarities, was used as input for the SIMPER tests.

The PERMANOVA, MDS, CAP, SIMPER analyses were performed using the routines included in the software PRIMER 6+^65,66^.

## Electronic supplementary material

Supplementary Information

## Data Availability

All data generated and analysed during this study are included in this published article and its Supplementary Information file.
