# Supplementary material for: Impact of mangrove forests degradation on biodiversity and ecosystem functioning
Source: Sci Rep. 2018 Sep 5;8:13298. doi: 10.1038/s41598-018-31683-0 (PMC6125342; doi:10.1038/s41598-018-31683-0)
Supplement: Supplementary file 1 — Supplementary Information [file 41598_2018_31683_MOESM1_ESM.docx]

**Impact of mangrove forests degradation on biodiversity and ecosystem functioning**

Laura Carugati^1,^*, Beatrice Gatto^1^, Eugenio Rastelli^1^, Marco Lo Martire^1^, Caterina Coral^1^, Silvestro Greco^2^ and Roberto Danovaro^1,2,^*

*^1^ Polytechnic University of March, e Department of life and environmental sciences, Ancona, 60131, Italy*

*^2^ Stazione Zoologica Anton Dohrn, Naples, 80121, Italy*

*^*^*Corresponding authors:

Roberto Danovaro: [r.danovaro@univpm.it](mailto:r.danovaro@univpm.it)

Carugati Laura: [l.carugati@univpm.it](mailto:l.carugati@univpm.it)

**LIST OF SUPPLEMENTARY INFORMATION**

**Supplementary Figure S1. Biochemical composition of sedimentary organic matter.** Reported are the concentrations of proteins (a), carbohydrates (b) and lipids (c) in undisturbed and disturbed mangrove areas. Reported are also average values of Undisturbed Mangrove (UM) and Disturbed Mangrove (DM) ± standard error.

**Supplementary Table S1.** Pairwise output to test differences among sites in organic matter.

**Supplementary Table S2.** Pairwise output to test differences among sites in meiofaunal variables.

**Supplementary Table S3.** Pairwise output to test differences among sites in prokaryotic variables.

**Supplementary information. Study area.**

| **Total phytopigment** | **Site** | t | **P(MC)** |
| --- | --- | --- | --- |
| **A) Undisturbed mangrove** | A, B | 1,202 | ns |
|  | A, C | 0,382 | ns |
|  | B, C | 1,338 | ns |
|  |  |  |  |
| **B) Disturbed mangrove** | A, B | 1,733 | ns |
|  | A, C | 2,620 | * |
|  | B, C | 2,328 | ns |
| **Biopolymeric C** | **Site** | **t** | **P(MC)** |
| **A) Undisturbed mangrove** | A, B | 1,183 | ns |
|  | A, C | 0,604 | ns |
|  | B, C | 0,227 | ns |
|  |  |  |  |
| **B) Disturbed mangrove** | A, B | 5,783 | ** |
|  | A, C | 6,368 | ** |
|  | B, C | 6,127 | ** |

**Table S1.** Pairwise tests for the concentrations of biopolymeric C and total phytopigment among sites sampled within the same area (t=statistic t for pairwise comparisons; P(MC)=probability level after Monte Carlo tests); **=P<0.01; *=P<0.05; ns=not significant.

| **Abundance** | **Site** | **t** | **P(MC)** |
| --- | --- | --- | --- |
| **A) Undisturbed mangrove** | A, B | 4,009 | ** |
|  | A, C | 1,529 | ns |
|  | B, C | 3,083 | * |
|  |  |  |  |
| **B) Disturbed mangrove** | A, B | 1,561 | ns |
|  | A, C | 0,429 | ns |
|  | B, C | 1,442 | ns |
| **Richness of higher taxa** | **Site** | **t** | **P(MC)** |
| **A) Undisturbed mangrove** | A, B | 2,673 | ns |
|  | A, C | 2,121 | ns |
|  | B, C | 1,414 | ns |
|  |  |  |  |
| **B) Disturbed mangrove** | A, B | 1,061 | ns |
|  | A, C | 4,000 | * |
|  | B, C | 0,378 | ns |
| **Composition as higher taxa** | **Site** | **t** | **P(MC)** |
| **A) Undisturbed mangrove** | A, B | 3,649 | ** |
|  | A, C | 2,422 | * |
|  | B, C | 3,028 | * |
|  |  |  |  |
| **B) Disturbed mangrove** | A, B | 1,238 | ns |
|  | A, C | 0,165 | ns |
|  | B, C | 1,045 | ns |
| **Composition as rare taxa** | **Site** | **t** | **P(MC)** |
| **A) Undisturbed mangrove** | A, B | 1,797 | ns |
|  | A, C | 1,905 | * |
|  | B, C | 1,016 | ns |
|  |  |  |  |
| **B) Disturbed mangrove** | A, B | 1,697 | ns |
|  | A, C | 1,227 | ns |
|  | B, C | 1,864 | * |

**Table S2.** Pairwise tests for meiofaunal abundance, higher taxa richness, taxonomic composition as higher and rare taxa among sites sampled within the same area (t, statistic t for pairwise comparisons; P(MC)=probability level after Monte Carlo tests); **=P<0.01; *=P<0.05; ns=not significant.

| **Abundance** | **Site** | **t** | **P(MC)** |
| --- | --- | --- | --- |
| **A) Undisturbed mangrove** | A, B | 6,404 | ** |
|  | A, C | 2,211 | ns |
|  | B, C | 1,782 | ns |
|  |  |  |  |
| **B) Disturbed mangrove** | A, B | 0,568 | ns |
|  | A, C | 3,372 | * |
|  | B, C | 3,482 | * |
| **Biomass** | **Site** | **t** | **P(MC)** |
| **A) Undisturbed mangrove** | A, B | 10,439 | ** |
|  | A, C | 4,505 | ** |
|  | B, C | 3,591 | * |
|  |  |  |  |
| **B) Disturbed mangrove** | A, B | 0,715 | ns |
|  | A, C | 18,516 | *** |
|  | B, C | 15,484 | *** |
| **Heterotrophic production** | **Site** | **t** | **P(MC)** |
| **A) Undisturbed mangrove** | A, B | 3,880 | * |
|  | A, C | 5,593 | ** |
|  | B, C | 2,951 | * |
|  |  |  |  |
| **B) Disturbed mangrove** | A, B | 2,029 | ns |
|  | A, C | 14,193 | *** |
|  | B, C | 1,889 | ns |

**Table S3.** Pairwise tests for prokaryotic abundance, biomass and heterotrophic production among sites sampled within the same area (t, statistic t for pairwise comparisons; P(MC)=probability level after Monte Carlo tests); ***=P<0.001; **=P<0.01; *=P<0.05; ns=not significant.

**Study area**

The investigated area is located at equatorial latitudes (1°45’ N, 125° 15’ E). The island counts 2,500 inhabitants (as of 2013), distributed throughout five main villages, four small resorts in the southeastern side, and a private research station hosting researchers and tourists. Based on the land use classification, the percentage of abandoned land is the dominant one (c.a. 22.6%). It is followed by orchads (c.a. 18.2%), shrubs and secondary forest (c.a. 14.1%) and coconut plantation (c.a. 14.1%). At time of sampling activities (2013), the investigated island had mangrove forests covering about 57.8 ha, equivalent to ca 2% of the total island surface. The dominant mangrove species belong to the genera *Avicennia*, *Rhizhopora*, and *Sonneratia*. Forest degradation and habitat loss have been occurring in many ways and over years on intense exploitation. The main impact observed on the degraded mangrove forests in this area include destructive fishing activities (e.g., blast fishing and poison fishing), the accumulation of marine litter and mining.
